# Are Signal Peptides Hidden Regulators of Neurodegenerative Disease?

**DOI:** 10.3390/biomedicines14081781

**Published:** 2026-08-07

**Authors:** Maciej Karbownik, Marcin Fidura, Renata Perlikowska

**Affiliations:** Department of Nucleic Acid Biochemistry, Medical University of Lodz, Pomorska 251, 92-213 Lodz, Poland; maciek.karbownik@gmail.com (M.K.);

**Keywords:** protein targeting, Alzheimer’s disease, Parkinson’s disease, Huntington’s disease, prion diseases, secretory pathway dysfunction, therapeutic targets, drug development

## Abstract

Canonical signal peptides (SPs) are short N-terminal sequences that direct nascent proteins into the secretory pathway, but their role extends far beyond protein targeting. Advances in sequencing and computational tools have enabled their systematic identification across proteomes, highlighting SPs as critical regulators of protein biogenesis, including endoplasmic reticulum (ER) targeting, translocation, folding, and proteostasis. Clinically, mutations affecting SP function underlie a distinct group of human disorders, while SP-derived fragments are emerging as diagnostic biomarkers and therapeutic targets. In biotechnology, SPs are engineered to enhance recombinant protein production and serve as molecular tags for intracellular delivery. Together, these developments position SPs at the intersection of fundamental cell biology, medicine, and biotechnology. While this review primarily focuses on canonical SPs, it also considers selected non-canonical targeting and topogenic sequences whose dysfunction contributes to protein misfolding, impaired ER translocation, disrupted degradation pathways, and altered intracellular trafficking in neurodegenerative diseases. Aberrations involving both conventional SPs and alternative targeting/topogenic elements contribute to pathological protein aggregation, a hallmark of major neurodegenerative disorders, including Alzheimer’s disease (AD), Parkinson’s disease (PD), Huntington Disease (HD), prion diseases, and amyotrophic lateral sclerosis/frontotemporal dementia (ALS/FTD); in multiple sclerosis (MS) is primarily an inflammatory demyelinating disease, where abnormal protein exposure, potentially linked to misprocessed SPs, can activate immune responses. By synthesizing current knowledge, the review explores how alterations in targeting determinants influence key proteostasis pathways, acting as upstream modulators of disease-relevant molecular cascades. It further discusses the emerging concept that SP-derived fragments may participate in intercellular communication, adding an additional layer of regulatory complexity.

## 1. Introduction

**Figure 1:**
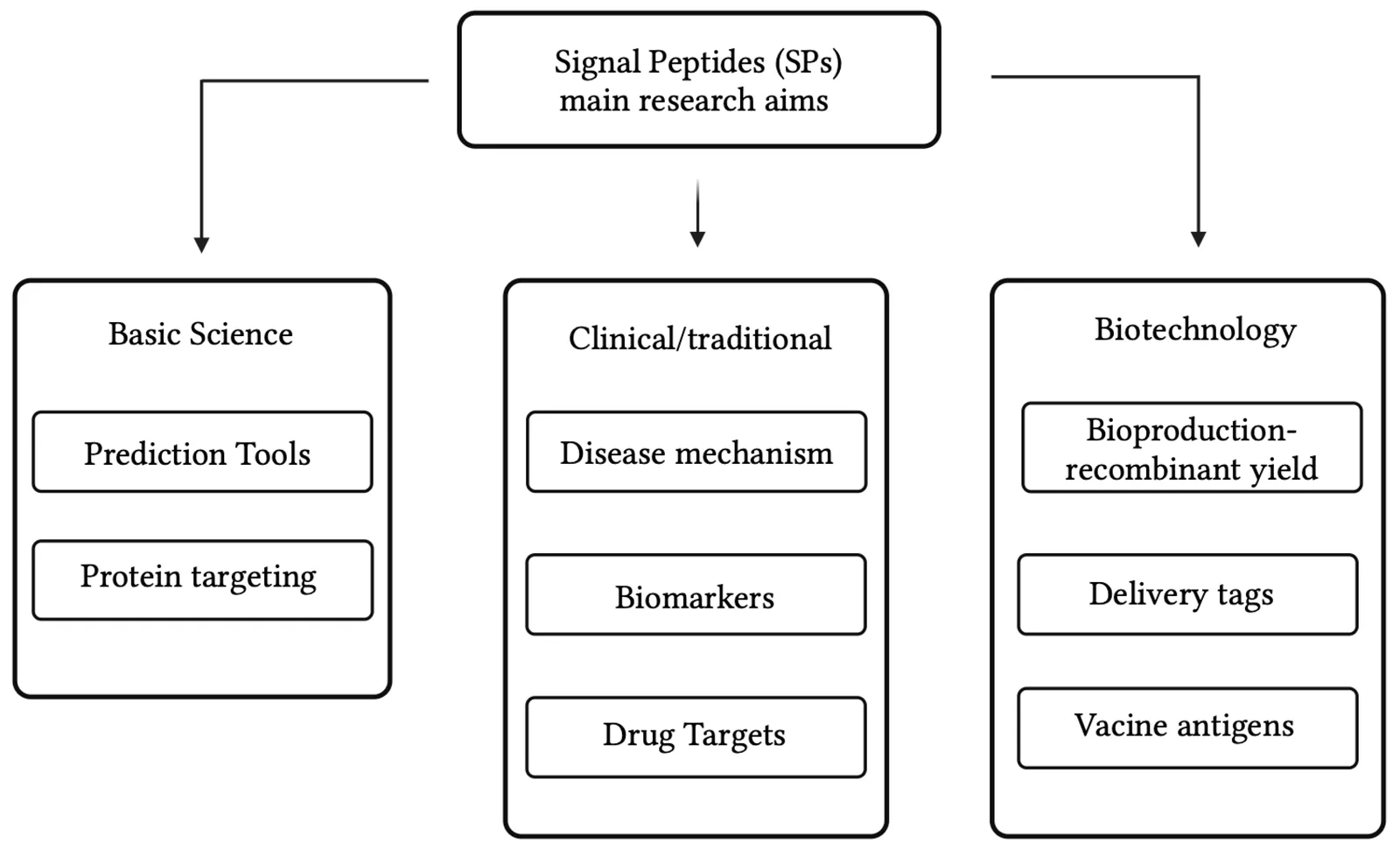
Signal peptides (SPs) across research domains: from basic science to clinical and biotechnological applications.

Signal peptides (SPs) are present across all prokaryotic and eukaryotic organisms. In the canonical view, they are short N-terminal sequences that play a pivotal role in directing newly synthesized proteins to the endoplasmic reticulum (ER) through the signal recognition particle (SRP)/Sec61-dependent secretory pathway. In the initial stage, they were regarded simply as postal codes that directed proteins to their destinations [1]; however, their detailed architecture was soon elucidated [2], and this breakthrough was ultimately recognized with the Nobel Prize awarded to Günter Blobel for the discovery of SPs [3]. In parallel with advances in biomedical sciences, SPs came to be recognized as multifaceted regulators of physiological processes, disease mechanisms, and therapeutic innovation [4]. Recent advances in molecular biology, bioinformatics, proteomics, and translational medicine have significantly expanded our understanding of SPs and their biomedical relevance. Thus, SPs have attracted significant attention in numerous scientific and industrial domains, including recombinant protein production, disease diagnostics, vaccine development, and various laboratory methodologies [5]. Together, these intersecting lines of research highlight SPs as an area of interest across fundamental cell biology, medicine, and biotechnology. Figure 1 provides an overview of the diverse roles of SPs, and together, these converging lines of research underscore their emerging importance.

Generally, SPs direct proteins to their site of action, and thereby help maintain intracellular homeostasis through the proper distribution of membrane proteins, receptors, neurotrophic factors, cytokines, and enzymes critical for cell growth, or survival. They are supposed to be neutral intermediaries, transient and rapidly degraded; however, when they are incorrectly produced, mutated, or inefficiently cleaved, ER targeting may be impaired and proteins can remain in the cytosol, where they misfold or aggregate [6,7], with downstream consequences that are shared with many other proteostatic insults rather than specific to SPs [8]. In a recent study, Thakur et al. [7] showed that the SP of human serum albumin (HAS, residues 1–18) can self-assemble into amyloid-like fibrils. By combining molecular dynamics simulations with biophysical and microscopic analyses, the authors reported that this SP can be amyloidogenic in vitro, raising the possibility that some SP sequences may themselves contribute to protein aggregation and cellular proteotoxicity.

Neurons are particularly vulnerable to disturbances in proteostasis, as their long-lived nature and high metabolic activity demand precise control of protein folding, trafficking, and degradation. Consequently, failure of proteostasis mechanisms is strongly linked to the onset and progression of neurodegenerative diseases [9]. A central component of this network is the ER, whose proper function is essential for neuronal survival [10,11]. Disruption of ER homeostasis leads to the accumulation of unfolded or misfolded proteins, triggering ER stress and activation of the unfolded protein response (UPR). While the UPR initially acts as an adaptive mechanism to restore proteostasis, its chronic activation or failure ultimately promotes cellular dysfunction and apoptosis. Such disturbances in ER proteostasis are well-established pathogenic mechanisms in major neurodegenerative disorders, including Alzheimer’s disease (AD), Parkinson’s disease (PD), Huntington’s disease (HD), and amyotrophic lateral sclerosis (ALS), characterized by the progressive accumulation of misfolded or aggregation-prone proteins, such as amyloid-β and tau aggregates in AD, α-synuclein in PD, mutant huntingtin in HD, and TDP-43 in ALS [12]. Multiple sclerosis (MS) represents a distinct case, primarily an inflammatory demyelinating disorder rather than an aggregation proteinopathy; it is included here because a coding variant in the SP of interleukin-22 binding protein (IL-22BP) impairs the protein’s secretion and thereby alters immune regulation. Whereas the studies cited above [9,10,11,12] address proteostasis mechanisms that act after protein misfolding has occurred, including the UPR, autophagy, and protein degradation, the present review focuses on the earlier step of SP-mediated ER targeting and translocation. As the first committed stage of the secretory pathway, specified by short, defined signal sequences and a limited set of processing enzymes, this process may represent, in principle, a more accessible point of therapeutic intervention than the broadly acting downstream stress responses.

**Figure 2:**
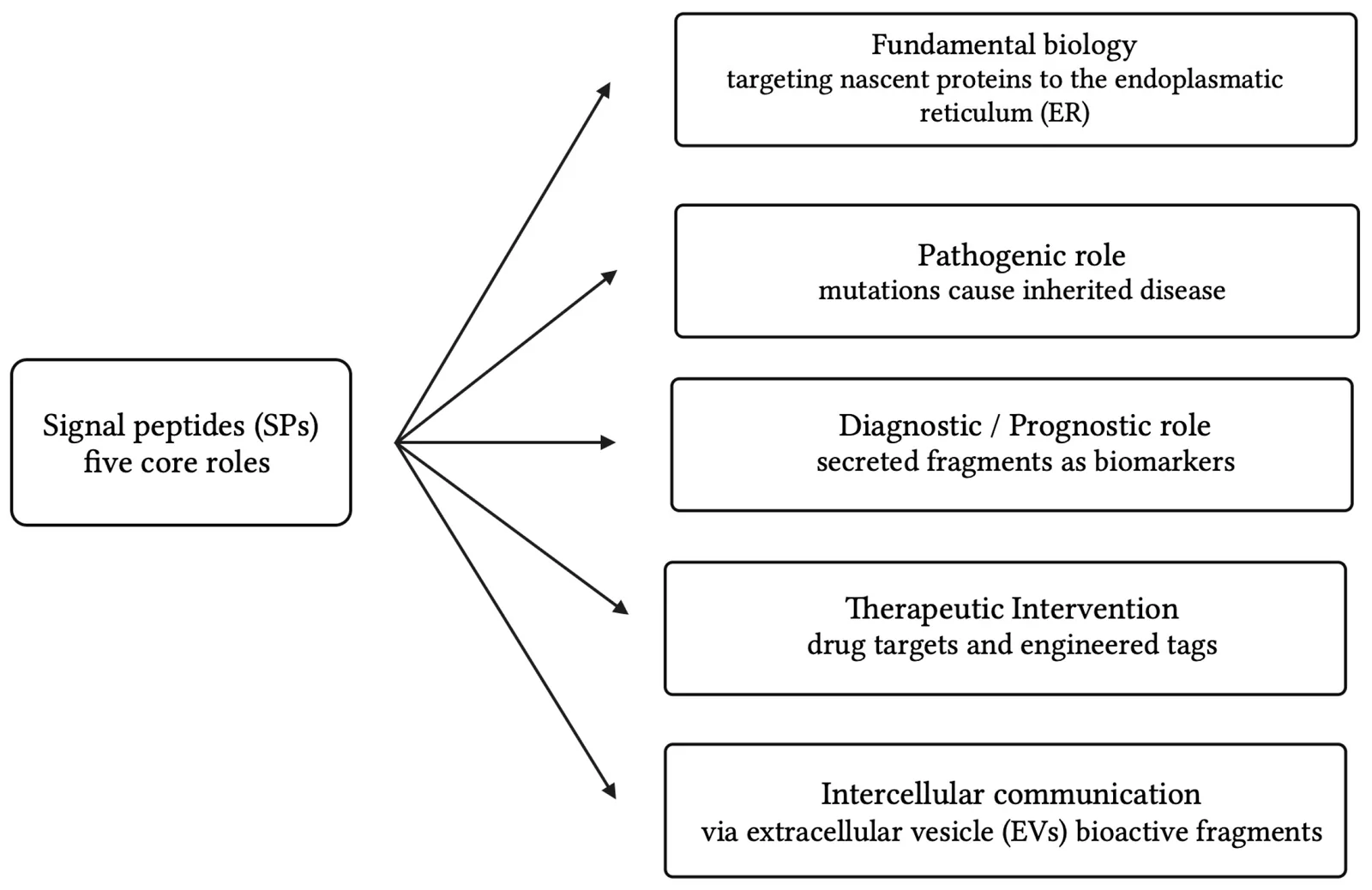
Functional roles of SPs: from their canonical function in ER targeting to emerging roles in pathology, biomarker discovery, therapeutic engineering, and cell–cell signaling.

Within this context, SPs, as the element that governs entry into the secretory pathway, is a plausible but largely untested point at which this network could be perturbed. Mutations or dysfunctions affecting SP recognition or processing can impair ER targeting, alter protein stability, and disrupt normal maturation pathways. As a result, proteins may fail to enter the secretory pathway efficiently, leading to their misfolding or aberrant accumulation. These defects exacerbate ER stress and place additional burden on quality control systems, thereby promoting proteostasis collapse. Thus, although not primary pathological hallmarks, SP defects contribute to disease by linking impaired protein targeting to ER dysfunction, proteotoxic stress, and mechanisms underlying neurodegeneration [10,12,13]. To comprehensively characterize the role of SPs in neurodegenerative diseases, it is essential to consider their normal physiological functions, which are summarized in Figure 2.

At a fundamental biology level, SPs are central to protein biogenesis and cellular homeostasis, directing nascent polypeptides to the ER and regulating key processes such as translocation, folding, and secretion, thereby governing early stages of proteostasis [4]. The second major role of SPs relates to their involvement in disease pathogenesis [13,14]. Disruption of SPs function, particularly through mutations affecting targeting, cleavage, or intracellular trafficking, constitutes an important disease mechanism, resulting in ER stress, protein misfolding, and functional deficits or toxic protein accumulation. A third key aspect of SP biology is their diagnostic and prognostic potential, which, although still largely experimental, shows considerable promise [15]. SPs are gaining attention as emerging biomarkers, as their fragments can be detected in biological fluids including blood, cerebrospinal fluid, and extracellular vesicles, reflecting alterations in the secretory pathway and cellular stress states. In parallel, it is also important to consider their role in therapeutic intervention [4]. SPs are being explored as therapeutic targets and biotechnological tools, where engineered SPs can enhance protein secretion and improve recombinant expression systems, while SP-derived peptides are increasingly recognized as bioactive molecules capable of modulating protein aggregation and signaling pathways. Finally, SPs can be considered as regulators of intercellular communication, a recently emerging and high-impact area of research. Accumulating evidence indicates that SPs and their fragments can be released into extracellular vesicles, thereby linking intracellular proteostasis with extracellular signaling networks [15,16]. Together, these perspectives position SPs as multifunctional regulators at the interface of protein homeostasis, disease pathogenesis, and translational innovation.

Previous reviews have described SP structure and function [4,5], pathogenic SP variants across the human genome [13], the translational control of secretory proteins [14], and the emerging biology of SP-derived fragments [15,16]. The present review provides an SP-centered synthesis across multiple neurodegenerative conditions, and applies clear criteria to distinguish SP-specific effects from general disturbances in proteostasis. Overall, the evidence suggests that, in most disorders, SPs act as upstream modulators of disease-related pathways rather than as direct causes of disease. By affecting how efficiently proteins enter the secretory pathway, they can influence cellular vulnerability to protein-handling defects.

This review summarizes current knowledge on SPs in neurodegenerative diseases, focusing on their structural features and biological functions. It further discusses their roles in modulating the pathophysiology of neurodegenerative diseases, evaluates their potential as biomarkers for disease diagnosis and progression, and explores their relevance as emerging therapeutic targets.

## 2. Structural Fundamentals of SPs

From a historical perspective, the discovery of SPs was closely intertwined with efforts to understand how proteins reach their correct cellular destinations. Between 1971 and 1975, Blobel, Sabatini, and colleagues [1,2,3,17,18] showed that proteins carry intrinsic signals guiding their translocation and maturation, exemplified by the N-terminal cleavage of IgG within the ER. By 1983, the architecture of these targeting sequences, soon known as SPs, was elucidated [2], culminating in Blobel receiving the 1991 Nobel Prize for uncovering the fundamental principle of signal-mediated protein sorting [3].

Canonical SPs are short amino acid sequences located at the N-terminus of newly formed polypeptides that play a critical role in the precise trafficking of proteins to their appropriate cellular compartment or extracellular localizations [4,5]. It has long been believed that cleaved SPs are quickly degraded and processed near the ER. However, recent studies have shown that fragments of these peptides can be found in extracellular fluids, including the bloodstream, where they retain biological activity [15,16]. Moreover, SP fragments can be transported into extracellular environments via vesicular structures such as exosomes and microvesicles, which play a key role in intercellular communication. These observations indicate that SPs and their derivatives may perform functions that extend beyond their traditionally recognized roles [15].

SPs are typically 16–30 amino acids long and consist of three distinct regions: a positively charged N-region (~1–5 residues), a hydrophobic H-region (~7–15 residues), and a polar C-region (~3–7 residues), although their amino acid sequences are not conserved [5,19]. Such variability in amino acid composition was thought to enable efficient translocation, and SPs were considered highly mutation-tolerant and functional across distant heterologous hosts [5].

### 2.1. The N-Region

The N-region of an SP is usually made up of a short stretch of positively charged amino acids, mainly lysine (Lys), arginine (Arg), and histidine (His), since their positioning can strongly affect secretion efficiency. This region plays a critical role in the early stages of protein targeting and translocation, because, it facilitates interactions with negatively charged components such as the phospholipid bilayer, as well as protein factors involved in secretion, including the SRP, secretion protein B (SecB) chaperone, and SecA ATPase [5]. These interactions are essential for directing proteins toward the membrane translocation machinery. N-region length and charge vary across systems, being shorter and less positively charged in eukaryotes and longer and more charged in prokaryotes, especially Gram-positive bacteria [4,20,21]. This positive charge helps orient the SP correctly in the membrane, though an optimal balance is required; moderate charge enhances secretion, while excessive charge can impair it by causing aggregation or stress.

In both bacterial and eukaryotic systems, methionine (Met) commonly serves as the first amino acid of an SP. It associates with tRNA at the ribosome and recognizes the start codon (AUG) on mRNA, initiating protein synthesis [22,23]. In bacteria, Met is modified into formyl-methionine (fMet), which acts as the starting point for polypeptide formation [24]. In contrast, in eukaryotes, Met remains unmodified and directly participates in translation [22,25]. A typical N-region motif can be described as Met-Arg/Lys-X, although its length differs depending on the SP type. For instance, Tat (twin-arginine translocation) SPs possess a longer N-region compared to Sec SPs [26]. The positive charges within the N-region, together with the hydrophobic H-region and polar C-region, contribute to maintaining the correct topology of the polypeptide during its translocation [27]. These positive residues are especially important for the secretion of small eukaryotic proteins. As the SP directs the emerging peptide chain, electrostatic interactions with the negatively charged membrane facilitate its insertion. Subsequently, the SP is cleaved by signal peptidase I (SPase I), while remaining associated with the inner side of the plasma membrane [28].

### 2.2. The H-Region

The H-region constitutes the hydrophobic core of the SP and is capable of adopting an α-helical structure. Its primary role is to facilitate the targeting, insertion, and translocation of the nascent peptide across the cell membrane. Hydrophobicity is the key determinant of the H-region’s function, because it enables proper interaction between the SP and the membrane, its orientation, the efficiency of translocation, and even which secretion pathway a protein will follow (SRP, Sec, or Tat). A decrease in hydrophobicity often leads to reduced or completely inhibited secretion, whereas moderate increases can enhance protein export. However, excessive hydrophobicity can be harmful, causing protein aggregation, blockage of the translocation machinery, and reduced cell viability [5]. Thus, an optimal hydrophobic balance is essential. Generally, highly hydrophobic SPs tend to engage the SRP-dependent pathway, while less hydrophobic ones are directed toward the Sec pathway, and even lower hydrophobicity is characteristic of Tat-dependent substrates. In some cases, proteins with intermediate hydrophobicity may utilize more than one pathway. Moreover, the H-region contributes to processes such as folding and glycosylation of the emerging polypeptide chain. Typically, the H-region is composed of 7 to 15 hydrophobic amino acids, mainly leucine (Leu), isoleucine (Ile), and valine (Val) [29]. Among these, Leu is the most prevalent and often appears in a conserved pattern of three consecutive residues (Tri-Leu), especially in higher eukaryotes like mammals. In Gram-positive bacteria, a highly conserved YSIRK/G-XX-S motif has been identified at the beginning of the H-region in cell wall–anchored surface proteins [30]. This motif directs precursor proteins to specific regions of the cell membrane, indicating a role in spatial targeting and regulation. In organisms such as *Staphylococcus aureus*, secretory precursor proteins containing this motif are involved in coordinating lipoteichoic acid synthesis, which in turn influences the assembly efficiency of bacterial virulence factors [31].

### 2.3. The C-Region

The C-region, often referred to as the processing region, is situated at the C-terminus of the SP. It contains a loosely conserved cleavage site that is recognized by SPase, enabling accurate removal of the SP at a defined position. Within this site, residues upstream of the cleavage point are labeled P1 (−1) and P2 (−2), while those downstream are denoted P1′ (+1) and P2′ (+2). This cleavage step is essential for proper maturation and functional activation of the newly synthesized protein.

**Figure 3:**
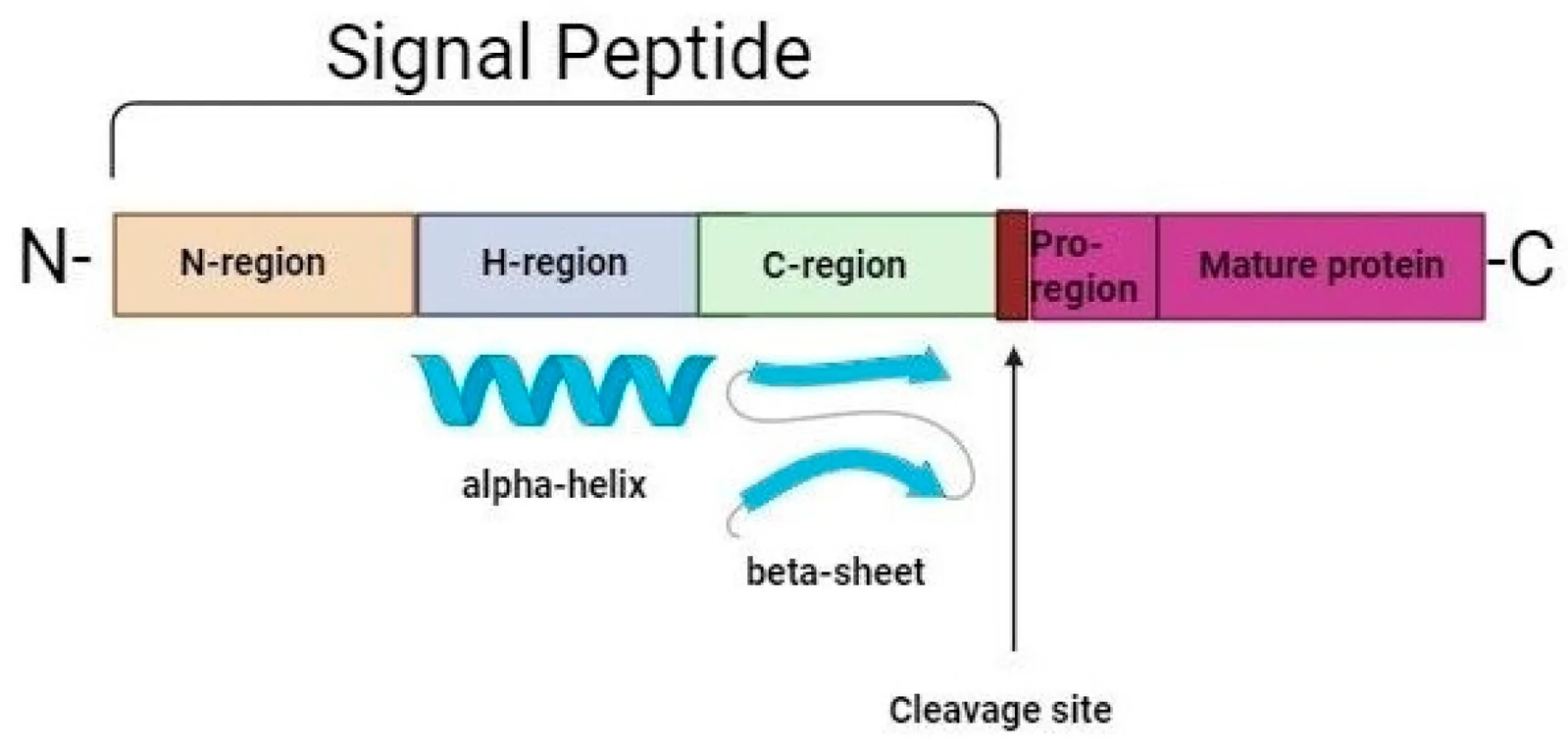
General architecture of canonical SPs, comprising the N-region (positively charged), H-region (α-helical), and C-region (cleavage site), followed by a pro-region preceding the mature protein.

Typically, the C-region is composed of 3–7 neutral or polar amino acids [29,32]. These residues possess hydrophilic side chains that stabilize the secondary structure through non-covalent interactions. The length, exact cleavage position, and degree of conservation of the C-region differ among various secretory proteins. In general, eukaryotic SPs have shorter C-regions than prokaryotic ones. Among bacteria, Gram-positive species tend to have longer C-regions (around 9–11 residues), whereas Gram-negative bacteria usually contain shorter ones (about 5–6 residues) [5,33]. The length of the C-region plays a key role in processing efficiency. Experimental modifications of the C-region in *B. stearothermophilus* amylase showed that optimal processing occurred with a length of five amino acids. Increasing the length led to reduced processing efficiency, and when extended to 13 residues, cleavage was completely inhibited [33,34]. The general architecture of SP is presented in Figure 3.

These three regions of SPs function together as an integrated unit to ensure efficient protein targeting and secretion. The N-region initiates interactions with the cytosolic side of the membrane and helps establish correct orientation, the H-region anchors the peptide within the membrane and largely determines pathway selection through its hydrophobicity, and the C-region ensures precise cleavage and release of the mature protein. Their coordinated properties, such as charge, hydrophobicity, sequence composition, and structure, collectively direct nascent proteins to the appropriate translocation machinery, enabling their proper delivery across or into membranes, including the ER in eukaryotes or the plasma membrane in prokaryotes. Their structural organization and mechanistic roles in co-translational targeting are detailed in recent molecular reviews [15].

### 2.4. The Pro-Region

It is worth noting that the pro-region, located just after the SP cleavage site, supports efficient protein secretion and proper folding. It typically contains neutral or negatively charged residues that help balance the positively charged N-region and facilitate membrane interaction. The first few residues (especially P1′) are critical for correct cleavage and must be small and flexible. Removing or altering this region often reduces secretion efficiency and leads to protein aggregation.

### 2.5. Computational Prediction of Canonical SPs

Modern bioinformatic tools, such as SignalP 6.0 (DTU Health Tech, Lyngby, Denmark), have enormously improved canonical N-terminal SP prediction accuracy, enabling detailed insight into their structure and function directly from amino acid sequences [35]. According to SignalP 6.0, SPs are categorized into five classes based on protein translocation pathway and SPase specificity: Sec/SPI (classical Sec-dependent peptides cleaved by SPase I), Sec/SPII (lipoprotein SPs containing a characteristic lipobox motif and processed by SPase II), Tat/SPI (Tat-pathway peptides with a conserved twin-arginine motif), Tat/SPII (Tat-dependent lipoprotein signals), and Sec/SPIII, which represent non-classical SPs, such as those found in type IV pilin precursors, often lacking a typical hydrophobic H-region [4]. This classification reflects both mechanistic and functional diversity, as SPs direct proteins through distinct pathways, including the Sec, SRP, and Tat systems, depending on their structural features. Characteristic sequence motifs further define these classes; for example, the lipobox motif in lipoprotein SPs enables membrane anchoring, while the twin-arginine (RR) motif is essential for Tat recognition.

Importantly, SignalP applies machine-learning approaches to achieve high prediction accuracy, allowing not only identification of SPs and their cleavage sites but also assignment to specific secretion pathways. This represents a major improvement over earlier motif-based methods, as it captures complex sequence patterns and contextual dependencies. Consequently, SignalP has become a powerful tool for proteome-wide annotation, functional classification, and recombinant protein design, supporting applications in biotechnology, vaccine development, and biomedical research.

### 2.6. Beyond Canonical SPs

While SignalP 6.0 represents the current gold standard for predicting canonical N-terminal cleavable SPs, several proteins contain non-canonical targeting domains, such as the N17 region of huntingtin (HTT), which functions as a membrane-associating and trafficking determinant as well as C-terminal glycosylphosphatidylinositol (GPI)-anchor signals that guide post-translational membrane attachment of proteins such as prion protein (PrP) [36]. In addition, a growing number of proteins involved in physiological and pathological signaling undergo unconventional secretion despite lacking classical SPs altogether. These non-canonical determinants and leaderless pathways fall largely outside the scope of current prediction algorithms.

In general, the non-classical signals are rather sequence motifs or domains that share functional features with SPs in directing protein localization and influencing its processing, despite differences in structure and cellular mechanism. Consequently, computational prediction of non-classical secretion and targeting signals remains considerably less developed than prediction of conventional SPs. In practice, many of these determinants cannot yet be identified from sequence alone and must be characterized experimentally. Thus, this limitation should be considered when interpreting bioinformatic analyses and when evaluating their translational potential. These different targeting mechanisms are best viewed together. Table 1 summarizes the major classes of canonical and non-canonical protein targeting signals found in proteins, highlighting their localization, processing, principal biological functions, and representative examples.

## 3. Role of SP Abnormalities in Disease

Having characterized the presence of SPs, their fundamental roles in cellular homeostasis, and their architecture, we now turn to the impact of their abnormalities in human disease, which can arise from mutations altering their primary structure, disrupting proper targeting and processing of nascent proteins. Beyond such genetic alterations, defects in SP recognition, cleavage efficiency, or interactions with the SRP and translocation machinery can also contribute to disease [13], although, as discussed below, the strength of this evidence varies considerably between disorders. Starting from the most well-recognized and extensively studied cases, mutations within SP sequences have been linked to disorders such as hemophilia A [37], familial neurohypophyseal diabetes insipidus [38], inherited retinal disorders [39], hypoparathyroidism [40], and skin diseases [41], often by impairing the hydrophobic core or cleavage of the SP, resulting in mislocalization or defective secretion of proteins. When the SRP fails to recognize a SP due to reduced hydrophobicity caused by mutations, it activates the quality control mechanism called regulation of aberrant protein production (RAPP), leading to selective degradation of the corresponding mRNA for secretory proteins, representing a novel molecular mechanism underlying various diseases [42]. A 2023 genome-wide study [13] demonstrated that the majority of pathogenic variants of SP are concentrated within the hydrophobic core, while others occur in the N-terminal region or at the SPase cleavage site, providing a useful framework for understanding disease mechanisms in both secreted and membrane proteins.

These monogenic disorders are useful not only as examples but also as a benchmark for strong evidence, because in each case, three conditions are met. First, the disease-causing mutation lies in the SP, not in the mature protein. Second, experiments show that the SP itself causes the defect. This is demonstrated when the mutant SP reproduces the trafficking problem after being attached to another protein, or when replacing it with a normal SP restores secretion. Third, the effects are specific to the affected protein rather than a general consequence of cell stress.

This is important because many diseases, including neurodegenerative disorders are characterized by protein misfolding, ER stress, and activation of the unfolded protein response [9,10,11,12]. Since these changes can occur whenever the secretory pathway is disrupted, their presence alone does not prove that the SP is involved. We already mentioned that neuronal survival critically depends on the maintenance of protein homeostasis, which requires tightly regulated processes of protein folding, trafficking, and degradation. Disruption of ER function leads to protein misfolding, ER stress, and prolonged activation of the unfolded protein response, contributing to neurodegeneration. In this context, defects in SP structure or processing can impair early steps of protein targeting and secretion, promoting protein misfolding, aggregation, and cellular stress, and thereby contributing to neurodegenerative disease pathology.

SP abnormalities play a genuine but often indirect role in disease, primarily what was already emphasized, by disrupting early stages of protein biogenesis before secretion or membrane insertion. These disturbances can lead to protein deficiency, mislocalization, intracellular retention or degradation, and in some cases altered immune recognition. To explore whether SPs act as hidden regulators of neurodegenerative disease, this section provides an overview of key neurodegenerative conditions while highlighting the emerging role of SPs in shaping their underlying molecular mechanisms. To better understand the potential role of SPs and their derived fragments in neurological disease, we consider a spectrum of disorders spanning protein misfolding and aggregation, including AD, PD, HD, and prion diseases, followed by mixed neurodegenerative conditions such as ALS/FTD, and finally neuroinflammatory disorders such as MS.

### 3.1. Protein Misfolding and Aggregation: The Role of SPs in AD

AD is characterized by the progressive accumulation of extracellular amyloid-β (Aβ) plaques and intracellular neurofibrillary tangles composed of hyperphosphorylated tau, leading to synaptic dysfunction and neuronal loss. These pathological processes trigger neuroinflammation, oxidative stress, and impaired proteostasis, ultimately resulting in cognitive decline and neurodegeneration. Amyloidogenic processing of amyloid precursor protein (APP), leading to the production of Aβ peptides, is a key mechanism driving AD. Misprocessing of APP, particularly increased production of Aβ42, a major driver of plaque formation. The regulation of amyloidogenic APP processing is complex and involves numerous intracellular pathways. Several molecular regulators are involved in shifting APP processing toward the amyloidogenic pathway, including: β-secretase (BACE), furin, hypoxia-inducible factor 1 alpha (HIF-1α), alcadeins, prion protein (PrP), α-synuclein (α-Syn) [43]. These regulators can modulate secretase activity, influence APP trafficking, or alter APP cleavage dynamics. The interplay between these regulatory proteins suggests that AD pathology arises not from a single defect but from multiple converging disturbances in cellular homeostasis. The shifts in the balance between amyloidogenic and non-amyloidogenic processing influence whether APP is cleaved into harmless products or pathogenic Aβ. While studies traditionally focus on Aβ generation, it has been proposed that APP-derived fragments other than Aβ, including its SP, may contribute to disease processes [6]. In a single in vitro study, a synthetic peptide corresponding to the APP SP (residues 1–17) was shown to self-assemble into amyloid-like aggregates and to accelerate Aβ42 fibrillization, with associated cytotoxicity in SH-SY5Y cells and haemolytic activity in erythrocytes [6]. This observation is of interest because it indicates that SP fragments need not be biologically inert. A comparable propensity has subsequently been described for the unrelated SP of HAS (residues 1–18) [7], which raises the possibility that amyloidogenicity is a general biophysical property of short hydrophobic sequences rather than a specific feature of APP. Several important limitations should nevertheless be emphasized. The findings derive from synthetic peptides studied at micromolar concentrations in cell-free and cell-culture systems; it is not known whether comparable local concentrations of the free SP are reached in vivo, nor whether the SP and Aβ co-localize in the relevant subcellular compartments. No genetic, neuropathological, or in vivo evidence currently links the APP SP to amyloid pathology, and the finding has not been independently replicated. A contribution of the APP SP to AD therefore remains a hypothesis rather than an established mechanism.

### 3.2. PD Without Canonical SP Involvement

PD is a progressive neurodegenerative disorder marked by the degeneration of dopaminergic neurons in the substantia nigra and the accumulation of α-Syn aggregates (Lewy bodies). These pathological changes lead to motor symptoms such as tremor, rigidity, and bradykinesia, as well as non-motor dysfunction. The central protein in PD, α-Syn is fundamentally defined by the absence of a classical N-terminal SP, to target it to the ER for secretion, what is more the process by which leaderless proteins reach the plasma membrane and extracellular space, such as cerebrospinal fluid, and blood plasma, is collectively termed “unconventional secretion” and bypassed the canonical ER-Golgi pathway. Alpha-synuclein (α-Syn) is secreted via several pathways, and the best-characterized route is misfolding-associated protein secretion (MAPS), which enables the extracellular release of misfolded cytosolic proteins. It can also be secreted in exosomes, transferred between cells through tunneling nanotubes, or released via ER/Golgi-independent vesicular exocytosis, demonstrating that its extracellular spread occurs entirely through signal peptide-independent mechanisms [15,44,45]. Secretion of α-Syn is markedly enhanced under conditions of cellular stress, including impairment of the proteasome, lysosomal/autophagic dysfunction, and ER stress. This suggests that extracellular release may function as a protective “overflow” mechanism, allowing cells to eliminate excess or misfolded protein when intracellular degradation pathways are compromised.

While α-Syn lacks its own SP, researchers have exploited SPs experimentally to study disease spread [46]. They engineered α-Syn by fusing it to a SP, thereby promoting its active secretion from neurons. This construct was delivered to the brains of wild type, non-transgenic mice, enabling controlled production of extracellular α-Syn in vivo. This study shows that neuronal secretion and intercellular (cell-to-cell) transmission of α-Syn are sufficient to drive widespread PD-like pathology in vivo, highlighting extracellular α-Syn as a key pathogenic mediator. Once released into the extracellular space, α-Syn can be internalized by neighboring neurons, where it acts as a template for misfolding and aggregation of endogenous protein. This process enables intercellular propagation of pathological α-Syn, resembling a prion-like mechanism of transmission. Such propagation is thought to play a central role in disease progression, as it facilitates the spread of toxic aggregates across anatomically connected neuronal networks. Indeed, this mechanism is considered to underlie the progressive dissemination of Lewy pathology observed in PD, consistent with the Braak staging hypothesis, which describes a sequential and region-specific spreading of α-Syn pathology throughout the nervous system [47,48,49]. Therefore, experimental fusion of a heterologous SP to α-Syn, which directs it into the conventional secretory pathway, alters its intracellular trafficking and pathological properties [46], indicating that the route of cellular exit is not merely incidental but has a significant impact on the resulting biological outcome. Thus, PD serves as a mechanistic counterpoint rather than an example of SP dysfunction.

### 3.3. Signal-like Domains in HD

HD is a rare inherited neurodegenerative disorder that progressively damages nerve cells in the brain, leading to movement problems, cognitive decline, and psychiatric symptoms. It is caused by a CAG trinucleotide expansion (36 or more repeats) in the huntingtin (HTT) gene on chromosome 4. In healthy individuals, the HTT gene produces huntingtin (HTT), a large, multifunctional protein essential for neuronal health. It supports embryonic development, protects neurons, regulates gene expression, enables efficient axonal transport and intracellular signaling, and maintains proper vesicle trafficking. In HD, the HTT gene’s CAG repeat expansion produces a mutant huntingtin protein that misfolds, aggregates, disrupts essential cellular functions, loses its neuroprotective roles, and ultimately causes progressive neurodegeneration in the brain. There is no evidence that HTT involves classical SPs (N-terminal ER-targeting SPs) for the secretory pathway. HTT is a cytosolic protein; thus, instead, it contains a 1–18 amino acids N terminal membrane association signal-like domains that regulate localization, trafficking, and aggregation [50]. These N-terminal motifs form an amphipathic α-helical membrane-binding signal and are recognized as the N17 domain, precisely 17 amino acids at the N-terminus of the huntingtin exon 1 fragment, sitting immediately before the polyglutamine (polyQ) repeat [51,52]. The N17 domain of mutant huntingtin exon 1 (mHTTEx1) plays a central role in driving pathogenicity through multiple, interconnected mechanisms. Owing to its amphipathic α-helical structure, N17 promotes aggregation by nucleating misfolding events that are subsequently propagated by the expanded polyglutamine tract. This same structural property enables association with cellular membranes, thereby influencing both subcellular localization and aggregation propensity. In addition, N17 serves as a hub for post-translational modifications (PTM), such as phosphorylation and acetylation, which modulate aggregation dynamics and cellular toxicity [51,52]. Finally, N17 functions as a nuclear export signal, regulating the nucleocytoplasmic distribution of mHTTEx1 and thereby critically shaping its toxic potential. Because the N17 domain lies at the intersection of aggregation, membrane association, post-translational modification, and intracellular trafficking, it represents an attractive therapeutic target. Accordingly, current strategies aim to modulate N17 function to attenuate aggregation and slow HD progression by blocking its aggregation-driving properties, modifying its interactions with membranes and proteins, and controlling HTT localization to reduce toxicity [50,51,52,53,54]. Conceptually, this system aligns with other proteostasis disorders, because N17 acts as an intrinsic regulatory module that determines whether, where, and to what extent mutant huntingtin aggregates. In this sense, it parallels targeting and processing motifs such as SPs, although here the “signal” governs aggregation dynamics and nucleocytoplasmic trafficking rather than ER targeting.

### 3.4. Non-Canonical Signal Sequence in Prion Disease

Prion diseases are fatal neurodegenerative disorders caused by the conversion of normal, soluble and non-toxic cellular prion protein (PrPC) into a misfolded, aggregation-prone isoform, known as a scrapie prion protein (PrPSc) that propagates by templating further misfolding. This unique self-amplifying mechanism makes prion diseases transmissible, as well as sporadic or inherited (PRNP mutations). Accumulation of PrPSc leads to neuronal loss, spongiform degeneration, and rapid clinical decline, serving as a paradigm for protein-misfolding disorders. Although SPs are classically defined as N-terminal sequences directing proteins to the ER, PrPC also contains a C-terminal glycosylphosphatidylinositol (GPI)-anchor signal sequence that functions as a distinct targeting signal, guiding post-translational membrane anchoring and critically influencing protein maturation and neurotoxicity [55]. Thus, C-terminal “signal sequence” for GPI anchoring, is functionally similar to canonical SPs, but mechanistically distinct. Comparison of canonical N-terminal SPs and the C-terminal GPI-anchor signal sequence in PrPC, highlighting their distinct roles in targeting and post-translational processing (Table 2).

Mutations in the C-terminal (GPI)-anchor signal sequence of PrPC profoundly influence neurotoxicity by altering protein trafficking, membrane anchoring, and post-translational processing, thereby modulating disease onset, progression, and neuropathological phenotype. Puig et al. [56] investigated the role of the GPI-anchor signal sequence in PrPC by generating a chimeric protein with that of Thy-1, known as a neuronal cell surface glycoprotein tethered, both in cultured cells and in transgenic mice. This modification altered the composition of the GPI anchor and consequently changed PrPC localization, reducing its shedding and shifting its distribution toward axons. Functionally, these changes led to delayed prion disease progression, reduced neuroinflammation, and altered signaling pathways, demonstrating that the GPI-anchor signal sequence critically regulates PrPC sorting, processing, and pathogenic mechanisms.

Since the missense variant from Met to Arg in codon 232 (M232R) of the prion protein gene is responsible for approximately 15% of cases of genetic prion diseases in Japanese patients, an investigation of the M232R substitution in the GPI-attachment SP of PrPC was conducted using a transgenic mouse model [57]. This mutation accelerated prion disease onset in a strain-dependent manner, without altering strain-specific histopathological or biochemical features. Notably, the GPI anchor attachment site remained unchanged; instead, the mutation reduced the hydrophobicity of the C-terminal SP, impairing ER translocation efficiency and leading to decreased N-linked glycosylation and GPI maturation. These findings demonstrate that subtle alterations in the C-terminal SP can disrupt early biosynthetic processing, promote misfolding-prone states, and enhance neurotoxicity.

Research study presented by Gu et al. [58] explained how certain genetic mutations linked to familial Creutzfeldt–Jakob disease (CJD) change the way the protein inserts into membranes, and favor a potentially neurotoxic membrane orientation, contributing to neurodegeneration. These mutations increase the stability and transport of GPI-SP-mediated post-translationally translocated PrPC to the plasma membrane, where it is linked to the lipid bilayer in C-transmembrane (CtmPrP) orientation. These findings suggest that a fraction of the PrPC becomes aberrantly inserted into the membrane, where it is relatively resistant to degradation. As a result, the protein adopts an abnormal orientation, giving rise to CtmPrP, which is considered potentially neurotoxic. This misoriented species can accumulate at the cell surface, thereby contributing to cellular dysfunction. Furthermore, it has been demonstrated that the GPI signal peptide (GPI-SP) of PrP exhibits dual functionality: it acts as an efficient co-translational ER translocation signal, yet functions relatively inefficiently in post-translational translocation when fused to an unrelated protein. This highlights the intrinsic versatility of the GPI-SP. Collectively, these findings reveal the existence of an alternative pathway for ER translocation of nascent PrP and provide important insights into the mechanisms by which mutations within the GPI-SP may promote neurotoxicity.

### 3.5. Mix Complex Pathology of ALS and FTD

ALS and FTD are now recognized as a continuum rather than distinct disorders [59,60]. This shift is supported by strong pathological, genetic, and clinical overlap [61]. Pathologically, both conditions are frequently characterized by cytoplasmic accumulation of TAR DNA-binding protein 43 (TDP-43). Genetically, they share key mutations, most notably the C9orf72 hexanucleotide repeat expansion. Clinically, overlap is also evident, as many ALS patients develop FTD-like cognitive or behavioral symptoms, while some FTD patients exhibit motor neuron disease [62,63]. In simplified terms, ALS primarily affects motor neurons, leading to muscle weakness and paralysis, whereas FTD mainly involves the frontal and temporal cortices, resulting in behavioral and language disturbances. However, both diseases converge on shared molecular mechanisms, including TDP-43 proteinopathy, disrupted RNA metabolism, and impaired proteostasis.

Importantly, several of these shared factors, such as TDP-43, fused in sarcoma (FUS), both RNA/DNA-binding proteins prone to aggregation in neurodegeneration, and C9orf72-derived dipeptide repeat proteins, are closely linked to defects in protein trafficking, nucleocytoplasmic transport, and protein homeostasis, providing a conceptual bridge to SP function and intracellular protein sorting.

In contrast to secretory proteins, major ALS-associated proteins, including TDP-43, FUS, both RNA/DNA-binding proteins prone to aggregation in neurodegeneration, as well as superoxide dismutase 1 (SOD1), an antioxidant enzyme that becomes toxic upon mutation and misfolding, are predominantly cytosolic or nuclear and therefore lack classical N-terminal SPs and do not undergo ER targeting [64]. However, emerging evidence indicates that cleaved SP fragments can be detected in extracellular fluids and may serve as potential biomarkers, suggesting a broader role for SP–derived molecules in neurodegenerative disease diagnostics [65]. The authors emphasized that several peptide biomarkers are derived from proteins that enter the secretory pathway via SP-dependent mechanisms (e.g., proenkephalin (PENK), secretogranin I (SCG1), apolipoprotein C-I (APOC1)). While SP function was not directly assessed, the altered abundance of these peptides suggests broader dysregulation of secretory pathway processes in ALS.

An intriguing observation concerns TDP-43, that contains a bipartite nuclear localization signal (NLS) mediating its import into the nucleus rather than into the SP-dependent secretory pathway [66]. In contrast to secreted proteins mentioned above (such as PENK, SCG1, or APOC1), which contain classical SPs, TDP-43 functions as a nuclear protein regulating the expression of numerous genes, including those encoding proteins involved in vesicular trafficking, synaptic function, and secretion. Consequently, disruption of TDP-43 localization due to impaired NLS function leads not only to its cytoplasmic aggregation, but also secondarily to dysregulation of genes encoding regulators of axonal transport and synaptic vesicle function (stathmin-2, *STMN2*; Munc13-1, *UNC13A*) [67]. In addition, defects in Rab1-dependent ER-to-Golgi trafficking and Golgi fragmentation have been reported in ALS [68]. Together, these findings indicate that TDP-43 dysfunction can broadly impair intracellular and vesicular trafficking, including processes linked to the secretory pathway, without directly affecting SP recognition or cleavage. Although SPs themselves were not detected in the Muqaku et al. dataset [65], the observed changes in peptide levels derived from secreted and secretory pathway-associated proteins suggest a functional perturbation of the entire secretory system. Thus, TDP-43 mislocalization may represent an upstream mechanism underlying the peptidomic changes observed in ALS, which are more likely to reflect altered gene expression and protein processing than defective SP function. However, future investigations are needed to determine whether early events such as SP recognition and cleavage contribute to these alterations.

FTD, by contrast, provides a case in which defects in SP-mediated targeting contribute directly to neurodegeneration. Among the major genetic causes of FTD, mutations in the progranulin gene (*GRN*) stand out as a clear and mechanistically well-defined link between SP dysfunction and disease [69]. Pathogenic *GRN* variants lead to an approximate 50% reduction in progranulin levels (haploinsufficiency) and account for approximately 5–10% of all FTD cases and up to ~22% of familial FTD, with high penetrance [70]. Progranulin is a secreted glycoprotein expressed in both the central nervous system and peripheral tissues, and its biological function depends critically on efficient passage through the secretory pathway. This process is initiated by an N-terminal SP, which directs the nascent polypeptide to the ER, ensuring its entry into the secretory pathway. A particularly instructive protein-level mutation is p.A9D in exon 1 of *GRN*, which introduces a negatively charged aspartate instead of alanine at position 9 into the hydrophobic core of the SP [71,72]. This substitution disrupts SP function by impairing SRP recognition and ER targeting, as supported by SignalP predictions showing markedly reduced SP efficiency compared to the wild type [73]. Functionally, the A9D mutation represents a distinct pathogenic mechanism. Unlike most *GRN* variants, which reduce progranulin levels via loss of transcript, A9D does not affect mRNA abundance but instead impairs secretion, leading to intracellular retention of the protein [71,72]. This results in functional haploinsufficiency, as extracellular progranulin levels are reduced despite ongoing synthesis, demonstrating convergence on a common endpoint of progranulin deficiency [74]. Progranulin deficiency links SP dysfunction to neurodegeneration, as reduced extracellular and lysosomal progranulin impairs lysosomal homeostasis, autophagy, and neuroinflammatory regulation, ultimately driving degeneration of the frontal and temporal lobes [75].

### 3.6. Immunopathology of MS and the Canonical Example of SP Dysfunction

Unlike the disorders considered so far, MS is not characterized by the accumulation of an aggregation-prone protein. It is a chronic autoimmune disorder of the central nervous system defined by inflammation, demyelination, neurodegeneration, and the connection to SP biology lies not in protein misfolding but in the regulation of a secreted immune modulator. A direct link between SPs and MS pathogenesis has been identified in the case of a rare coding variant (rs28385692) within the SP of *IL22RA2*, which encodes the IL-22 binding protein (IL-22BP) [76]. This variant impairs the efficiency of SP-mediated secretion, leading to reduced release of IL-22BP isoforms and contributing to MS susceptibility. Mechanistically, this represents a canonical example of SP dysfunction, where IL-22BP normally acts as a soluble antagonist that restrains IL-22–driven inflammation, and its decreased secretion shifts the balance toward a pro-inflammatory state, thereby promoting autoimmune demyelination. In contrast to this well-defined pathogenic role, the application of SPs as diagnostic biomarkers in MS remains largely exploratory. Nevertheless, it is important to recognize that many established MS biomarkers are secreted proteins detectable in cerebrospinal fluid or blood, inherently linked to SP–dependent trafficking [77]. Well-known biomarkers reflect alterations in the cellular secretome and, consequently, in SP–dependent trafficking pathways. This relationship highlights significant opportunities for future research aimed at understanding how disruptions in the secretory pathway contribute not only to disease mechanisms but also to the composition and reliability of biomarker profiles.

In parallel, extensive proteomic studies, such as that of Bodar et al. [78], have identified numerous disease-specific molecules in cerebrospinal fluid, including a 22-protein panel that improves the diagnosis and prognostic stratification of MS, collectively suggesting that SP-dependent secretion pathways may represent a promising yet underexplored diagnostic avenue [15]. Together, these observations place SPs in MS primarily within a pathogenic/risk framework, exemplified by *IL22RA2*, while their diagnostic potential remains an emerging but as yet unestablished strategy.

## 4. Discussion

Today, SPs are recognized as multifunctional molecular elements essential not only for directing nascent proteins to membranes but also for enabling recombinant protein production in commercially significant level [79], serving as drug targets [80], supporting gene-therapy strategies [81], acting as diagnostic biomarkers [82], contributing to vaccine design [83], explaining certain human diseases [13], and mediating membrane integration processes such as those involved in prion proteins [84], and many more [5].

To answer the question posed in the title, whether SPs act as hidden regulators of neurodegenerative diseases, it is important to evaluate the strength of the available evidence. Table 3 provides a summary based on this approach. First, a well-established mechanism, in which a disease-causing variant within an N-terminal SP disrupts protein targeting or secretion, has been demonstrated only in FTD (*GRN*) and MS (*IL22RA2*). In both cases, the evidence is supported by human genetic studies and functional experiments [67,68,69,70,71,72,73,74,75,76]. Second, in HD and prion disease, the relevant sequences are signal-like or non-canonical rather than classical SPs (the N17 domain and the C-terminal GPI-anchor signal, respectively) [50,51,52,53,54,55,56,57,58]. Although the mechanistic evidence is strong, it relates to signal-dependent protein processing more broadly and not specifically to ER targeting. Third, in AD, PD, and ALS, the link remains indirect and preliminary, and the findings may be explained by general disturbance in proteostasis [6,44,45,46,64,65,66]. The major aggregating proteins in these disorders do not contain SPs, and the strongest evidence, suggesting that the APP SP may itself be amyloidogenic, is based on a single in vitro study [6]. Overall, current evidence suggests that SPs may act as disease-modifying factors in some neurodegenerative disorders, but their contribution varies considerably between diseases and they cannot yet be considered a common cause of neurodegeneration.

Is this evidence sufficient to consider SPs as potential therapeutic targets or biomarkers? Therapeutic interest in SPs has emerged from the observation that altered SP structure, aberrant cleavage, or dysregulated SP-dependent trafficking contributes to diverse diseases, including cancer, metabolic disorders, endocrine dysfunction, and neurodegeneration. Because SPs determine the efficiency, fidelity, and spatial routing of protein secretion, targeting SP-associated mechanisms enables modulation of entire secretory programs, rather than single downstream effectors. Among emerging therapeutic approaches, inhibition of SPase represents a key strategy, particularly in conditions where pathogenic proteins rely on SP-dependent maturation. SPase inhibitors, such as arylomycin-derived compounds, have shown antimicrobial efficacy by disrupting secretion of virulence factors [85]. Beyond antibacterial applications, targeting the secretory pathway is emerging as a broader therapeutic concept, especially in cancer, where elevated secretory demand creates vulnerability [86,87], and in viral infections that exploit host ER machinery [4,88].

Conversely, enhancing SP function represents an alternative therapeutic approach in diseases associated with protein misfolding or secretion defects. Engineering SP sequences through mutagenesis, sequence swapping, or machine learning-guided design has significantly improved protein secretion efficiency across multiple systems [89,90]. These findings establish SPs as tunable regulatory elements rather than passive targeting signals.

In the area of neurodegenerative diseases, Signal Peptide Peptidase-Like 2b (SPPL2b), demonstrate substantial translational potential in AD, based on convergent preclinical evidence. It is an intramembrane aspartyl protease, expressed in the hippocampus and cortex, regulates amyloid precursor protein cleavage through its substrate BRI2 and promotes neuroinflammation via TNFα cleavage [91,92,93]. Several peptidase inhibition strategies have been explored with varying efficacy, including genetic deletion, pharmacological approaches, and cell-penetrating peptide-based interventions. Genetic deletion of SPPL2b consistently reduced APP cleavage, as well as Aβ40 and Aβ42 production, decreased brain amyloid plaque deposition, and significantly reduced cortical and hippocampal gliosis at early disease stages across multiple independent studies [93,94,95]. Pharmacological inhibition studies have shown that γ-secretase inhibitors can effectively suppress SPP/SPPL activity by exploiting the shared catalytic motifs with presenilins [96]. Importantly, compounds (DAPT, LY-411757, NVP-AHW700-NX, L-686,458, (Z-LL)_2_-keton) displaying differential specificity toward each protease have been identified [96], underscoring the feasibility of developing selective inhibitors capable of reducing Aβ generation without interfering with other intramembrane aspartyl proteases. Additionally, in silico screening approaches have been used to identify potential SPPL2b inhibitors; however, the limited availability of highly specific compounds remains a significant challenge in this field [95]. Another alternative strategy to reduce Aβ aggregation employed modified SPs with cell-penetrating properties [97]. Notable examples include peptides derived from transthyretin (TTR), such as TTR 102–117, TTR 74–83, TTR 1–21, PrP 23–28, which exhibit anti-inflammatory properties and the ability to cross the blood–brain barrier. Importantly, these did not affect cell viability in serum-containing media, indicating a favorable safety profile at the tested concentrations. Moreover, SPPL2b exhibits biomarker potential through biphasic expression, early upregulation that enhances Aβ production in a vicious cycle, followed by downregulation correlating with synaptic loss in late pathology, suggesting utility for disease staging and patient stratification [94]. These preclinical findings are encouraging but require cautious interpretation. The pharmacological data rely largely on γ-secretase-directed compounds, a class that has failed clinically in AD. No selective SPPL2b inhibitor has yet been validated or tested in human neurons.

There is growing evidence supporting the role of SPs as biomarkers [4,15]. Clinically, SP defects are linked to distinct, measurable phenotypes. Mutations in SPs disrupt protein targeting and secretion, causing monogenic diseases such as early-onset diabetes (preproinsulin) [98], growth hormone deficiency (GHRHR) [99], hemophilia B (Factor IX) [100], and Leydig cell hypoplasia (LHCGR) [101,102], as well as influencing prion disease progression [58]. These variants serve as precise genetic biomarkers informing disease classification and prognosis.

However, despite this promise, no SP-derived biomarker has yet been FDA-approved, and their clinical application remains largely experimental [103]. Nevertheless, cardiovascular studies highlight their potential: while CNPsp does not support acute diagnosis, elevated levels correlate with mortality and reinfarction risk, underscoring the value of SP fragments as prognostic indicators [104,105]. The most advanced work on SP fragments as biomarkers focuses on prepro-atrial natriuretic peptide signal peptide (ANPsp; AA 16–25 and 18–25) [106]. These fragments are measurable in plasma using novel assays and show clear clinical potential. In patients with ST-elevation myocardial infarction, ANPsp levels rose earlier than cardiac troponin and peaked within ~5 h of symptom onset, supporting its value for early diagnosis of acute myocardial injury. Detectable baseline levels and observed arteriovenous gradients across the heart and kidneys further indicate physiological relevance and organ-specific handling.

In neurodegenerative disorders, SP fragments originating from membrane and secreted proteins also exhibit biomarker-like behavior. Fragments derived from the APP SP, including both C-terminal and N-terminal portions, have been shown to be secreted extracellularly via exosomes [15]. Their presence in circulation suggests that changes in neuronal secretory processing, an early hallmark of AD, may be traced by profiling SP fragment. Although the C-terminal fragment of the APP SP has been detected in exosomes released from cultured cells, showing that such fragments can be generated and packaged into extracellular vesicles rather than being fully degraded [15], this does not establish that they are amyloidogenic or pathogenic under physiological conditions. Similarly, N-terminal SP fragments from secreted alkaline phosphatase (SEAP) accumulate in exosomes during TLR3-mediated immune activation, demonstrating how neuroinflammatory or stress-responsive states may modulate SP fragment release [107]. Together, APP SP and SEAP SP fragments illustrate that SP-derived peptides can serve as extracellular reporters of disturbed vesicular trafficking, ER stress, or neuroimmune dysregulation [15].

### Limitations and Future Directions

A fundamental limitation of our work is that it is a narrative review. The available evidence is markedly heterogeneous, as summarized in Table 3, which encompasses human genetic associations supported by functional analysis through to single in vitro observations. Moreover, a substantial part of it derives from overexpression systems, the use of synthetic peptides, or transfected, non-neuronal cell lines rather than from neurons or in vivo models. We would emphasize that SPs are additionally difficult to study directly, because their rapid cleavage and degradation mean that their behavior is usually inferred from the fate of the mature protein. Computational prediction of SP function does not establish pathogenicity, and consequently most of the reported SP variants remain variants of uncertain clinical significance. A further limitation is inter-species differences, exemplified by the divergent consequences of TDP-43 depletion for the progranulin–sortilin axis [69,70,73]. Finally, the disorders considered here were selected on the basis of the availability of mechanistic data, and the contribution of SP variation to neurodegenerative risk at the population level has not yet been systematically assessed.

These limitations point to fairly specific directions for future research. One aim would be to establish whether a given defect resides in the signal sequence or in the mature protein. Moreover, large-scale population studies could show how frequently variants occur in signal sequences and whether these changes are more common in patients than in healthy individuals. This information is currently missing for almost all of the diseases discussed here, but it is essential for determining how much signal-sequence variants contribute to disease risk in the general population. In addition, determining whether the effects observed in transfected cells also occur at physiological expression levels will require knock-in rather than overexpression models, as well as patient-derived neurons. This applies with particular force to the reports of amyloidogenicity of the APP SP, which require independent confirmation before they can be regarded as established. It is also important to assess whether SP-derived fragments persist in human body fluids at concentrations consistent with a biological role, which is a prerequisite for their proposed use as biomarkers. Finally, if SPs act principally as upstream modulators, the most direct test of this hypothesis is whether common SP variants modify age at onset or penetrance in existing neurodegenerative disease cohorts.

## 5. Conclusions

Collectively, current evidence identifies SPs as an underappreciated regulatory layer in neurodegenerative and neuroinflammatory diseases, but one whose contribution is unevenly documented. When a disease-associated variant occurs within a functional SP and its effects have been experimentally confirmed, as in *GRN* in FTD, *IL22RA2* in MS, and *PRNP* M232R in inherited prion disease, the mechanism is relatively well understood [67,68,69,70,71,72,73,74]. In these cases, disruption of protein targeting or secretion leads to loss of protein function or altered immune regulation. Elsewhere the link is weaker, resting on signal-like domains rather than true SPs (in HD), on a single in vitro observation (the APP SP in AD), or on indirect proteomic association (in ALS). This gradient of evidence must be kept in view. Otherwise SPs are easily over-interpreted as a general driver of neurodegeneration.

Translationally, SPPL2b inhibition remains the most promising target in this area [93,94,95,96], but its development is constrained by the absence of selective inhibitors, limited dose–response and long-term safety data, and minimal validation in human systems. The biphasic expression profile further implies that any intervention will need to be stage-specific, and cell-penetrating SP-derived peptides remain unvalidated in vivo.

As biomarkers, SP fragments are limited by rapid co-translational cleavage, and whether advances in proteomics and liquid biopsy can overcome this remains to be seen. SP engineering, by contrast, is already a mature technology and currently represents the most immediately applicable aspect of SP biology.

In conclusion, SPs should no longer be viewed solely as passive address labels, but rather as active regulators whose perturbation can initiate or modulate neurodegenerative disease processes.

## Figures and Tables

**Table 1:** Major classes of canonical and non-canonical signals directing protein targeting, membrane association, and secretion.

Signal Type	Location	Cleavage	Main Function
Canonical SP	N-terminus	Yes	ER targeting via SRP/Sec61 pathway (e.g., amyloid precursor protein, APP)
Signal-anchor sequence	Usually N-terminus/internal	No	Membrane insertion and topology determination (e.g., Type II membrane proteins)
Signal-like domain	Variable	Usually no	Membrane association or trafficking (e.g., N17 region of huntingtin (HTT))
GPI-anchor signal	C-terminus	Processed during GPI attachment	Membrane anchoring (e.g., prion protein (PrP))
Unconventional secretion motif	Variable	Usually no	ER/Golgi-independent secretion (e.g., various leaderless proteins)

**Table 2:** Comparison of N-terminal SP and C-terminal GPI-anchor signal.

Feature	N-Terminal SP	C-Terminal GPI-Anchor Signal Sequence
Position	N-terminus	C-terminus
Primary function	Targeting to the ER	Signal for GPI anchor attachment
Processing	Cleaved by signal peptidase	C-terminal sequence cleaved and replaced by GPI anchor
Type of signal	Classical SP	Non-canonical (GPI-anchor signal sequence)

**Table 3:** Signal and signal-like sequences implicated in neurodegenerative disease: type of sequence and nature of the supporting evidence.

Disease	Protein/*Gene,* Variant	Type of Signal Sequence	Nature of Evidence	Ref.
AD	APP SP/*APP*	Canonical N-terminal SP	Biochemical/biophysical in vitro (synthetic peptide); cytotoxicity (SH-SY5Y), haemolysis	[6]
PD	α-Synuclein/*SNCA*	None; leaderless; unconventional secretion	SP used only as an experimental tool to force secretion in vivo	[44,45,46]
HD	Huntingtin/*HTT*	Signal-like domain (N17: amphipathic helix, membrane association, nuclear export signal); not a true SP	Structural/biophysical, cell biological, PTM mapping	[50,51,52,53,54]
Prion disease	PrP (*PRNP*, variant p.M232R)	Canonical N-terminal ER-targeting SPs and non-canonical C-terminal GPI-anchor topogenic signal	Human genetics (~15% of genetic prion disease in Japan) + transgenic mouse models + cell biological translocation assays	[55,56,57,58]
ALS	TAR DNA-binding protein 43 (TDP-43)/fused in sarcoma (FUS)	None; (Nuclear localization signal (NLS) trafficking signals)	Indirect: peptidomics showing altered abundance of peptides derived from SP-dependent secreted proteins (PENK, SCG1, APOC1); SPs not directly assessed	[64,65,66]
FTD	Progranulin (*GRN*, variantp.A9D)	Canonical N-terminal SP (hydrophobic core)	Human genetics (familial FTD) + cell biological (impaired secretion, intracellular retention) + computational (SignalP)	[67,68,69,70,71,72,73,74,75]
MS	IL-22BP (*IL22RA2*, variantrs28385692, p.Leu16Pro)	Canonical N-terminal SP	Genetic association in large cohorts + functional secretion assays	[76]

## Data Availability

No new data were created or analyzed in this study. Data sharing is not applicable to this article.

## Abbreviations

The following abbreviations are used in this manuscript:

Aβ	Amyloid-β
AD	Alzheimer’s disease
ALS/FTD	Amyotrophic lateral sclerosis/frontotemporal dementia
ANPsp	Prepro-atrial natriuretic peptide signal peptide
APOC1	Apolipoprotein C-I
APP	Amyloid precursor protein
Arg	Arginine
BACE	β-secretase
CJD	Creutzfeldt–Jakob disease
CtmPrP	C-transmembrane prion protein
ER	Endoplasmic reticulum
fMet	Formylmethionine
FUS	Fused in sarcoma
GHRHR	Growth hormone–releasing hormone receptor
GPI	Glycosylphosphatidylinositol
GRN	Progranulin gene
HAS	Human serum albumin
HD	Huntington Disease
HIF-1α	Hypoxia-inducible factor 1 alpha
His	Histidine
HTT	Huntingtin
IL-22BP	Interleukin-22 Binding Protein
IL-22RA2	Interleukin-22 Receptor Subunit Alpha 2
Ile	Isoleucine
Leu	Leucine
LHCGR	Luteinizing hormone/choriogonadotropin receptor
Lys	Lysine
M232R	Missense variant from Met to Arg in codon 232
MAPS	Misfolding-associated protein secretion
Met	Methionine
mHTTEx1	Mutant Huntington exon 1
MS	Multiple sclerosis
NLS	Nuclear localization signal
PENK	Proenkephalin
PD	Parkinson’s disease
polyQ	Polyglutamine
PrP	Prion protein
PrPC	Cellular prion protein
PrPSc	Scrapie prion protein
PTM	Post-translation modifications
RAPP	Regulation of aberrant protein production
RR	Twin arginine
α-Syn	α-synuclein
SCG1	Secretogranin I
SEAP	Secreted alkaline phosphatase
Sec	Secretion protein
SP	Signal Peptide
SPase I	Signal peptidase I
SPPL2b	Signal peptide peptidase-like 2b
SRP	Signal recognition particle
Tat	Twin-arginine translocation
TDP-43	TAR DNA-binding protein 43
TTR	Transthyretin
UPR	Unfolded protein response
Val	Valine
